# Assessing the utility of deep neural networks in detecting superficial surgical site infections from free text electronic health record data

**DOI:** 10.3389/fdgth.2023.1249835

**Published:** 2024-01-08

**Authors:** Alexander Bonde, Stephan Lorenzen, Gustav Brixen, Anders Troelsen, Martin Sillesen

**Affiliations:** ^1^ Aiomic, Copenhagen, Denmark; ^2^ Department of Organ Surgery and Transplantation, Copenhagen University Hospital, Rigshospitalet, Denmark; ^3^ Department of Orthopedics, Copenhagen University Hospital, Hvidovre, Denmark; ^4^ Institute of Clinical Medicine, University of Copenhagen, Copenhagen, Denmark; ^5^ Center for Surgical Translational and Artificial Intelligence Research (CSTAR), Copenhagen University Hospital, Rigshospitalet, Denmark

**Keywords:** surgery, artificial neural network (ANN), natural language processing, surgical site infection, postoperative complication

## Abstract

**Background:**

High-quality outcomes data is crucial for continued surgical quality improvement. Outcomes are generally captured through structured administrative data or through manual curation of unstructured electronic health record (EHR) data. The aim of this study was to apply natural language processing (NLP) to chart notes in the EHR to accurately capture postoperative superficial surgical site infections (SSSIs).

**Methods:**

Deep Learning (DL) NLP models were trained on data from 389,865 surgical cases across all 11 hospitals in the Capital Region of Denmark. Surgical cases in the training dataset were performed between January 01st, 2017, and October 30th, 2021. We trained a forward reading and a backward reading universal language model on unlabeled postoperative chart notes recorded within 30 days of a surgical procedure. The two language models were subsequently finetuned on labeled data for the classification of SSSIs. Validation and testing were performed on surgical cases performed during the month of November 2021. We propose two different use cases: a stand-alone machine learning (SAM) pipeline and a human-in-the-loop (HITL) pipeline. Performances of both pipelines were compared to administrative data and to manual curation.

**Results:**

The models were trained on 3,983,864 unlabeled chart notes and finetuned on 1,231,656 labeled notes. Models had a test area under the receiver operating characteristic curves (ROC AUC) of 0.989 on individual chart notes and 0.980 on an aggregated case level. The SAM pipeline had a sensitivity of 0.604, a specificity of 0.996, a positive predictive value (PPV) of 0.763, and a negative predictive value (NPV) of 0.991. Prior to human review, the HITL pipeline had a sensitivity of 0.854, a specificity of 0.987, a PPV of 0.603, and a NPV of 0.997.

**Conclusion:**

The performance of the SAM pipeline was superior to administrative data, and significantly outperformed previously published results. The performance of the HITL pipeline approached that of manual curation.

## Introduction

Correct, granular, and validated surgical outcomes data is a critical resource for multiple stakeholders, including patients, surgical units, hospitals, and reimbursement organizations. As such, these data are being used to alter reimbursements, provide critical transparency about performance and to reduce postoperative morbidity and mortality through standard operating procedure (SOP) optimization (1). Outcomes are generally captured through structured administrative data such as reoperation- and diagnostic codes. This structured data format has several strengths including easy accessibility and inexpensive capture (2). It is readily computable and can be used for complication surveillance and for the development and validation of predictive algorithms. However, a large part of surgical outcomes data is only captured in unstructured free text format, and administrative data often lack sensitivity, positive predictive value, and temporal precision (3).

One way of addressing the shortcomings of administrative data is through the manual curation of free text in patient charts. This approach has been used with great success by clinical registries such as the American College of Surgeons-National Surgical Quality Improvement Program (ACS-NSQIP) (4). Although the program has been shown to be cost-effective, manual curation remains time-consuming, expensive, and prone to human error (5).

An alternative to the manual curation of free text is the use of machine learning (ML) methods such as Natural Language Processing (NLP). NLP is a subfield of artificial intelligence (AI), where algorithms are trained to efficiently parse and analyze human language (6), thus offering the potential for NLP algorithms to automate chart reviews of surgical patients while addressing many shortcomings of both administrative data and manual curation.

The concept has previously been assessed across several postoperative complication subtypes with varied approaches and results. Most studies have deployed legacy approaches (7), whereas novel ML methods such as deep learning (DL) have only been deployed in a single study to date (8). DL models employ multi-layered neural networks and generally continue to improve with more training data, often outperforming classical ML methods (6). This has been highlighted in a recent meta-analysis of ML to capture surgical outcomes data, which stated: “Implementing deep learning NLP technology in surgery may be momentous in our ability to capitalize on free text analysis” (9).

The objective of the current study was to assess the value of DL NLP approaches to capture surgical outcomes data from medical free text and to compare the performance with current registration practices. To limit the scope of this study and provide proof of concept of the prospect of using DL NLP for postoperative phenotype detection, we chose to focus on one postoperative complication, namely superficial surgical site infection (SSSI). This choice stems from a previous study where surgical site infections were found to have the largest degree of administrative undercoding (10) but have a recognized clinical impact and importance. According to the Center for Disease Control (CDC) National Surveillance System, surgical site infections (SSIs) are classified as either superficial incisional SSI, deep incisional SSI and organ/space SSI. These occur near the surgical site within 30 days following surgery. Incisional/superficial SSI's involve only the skin and subcutaneous tissue (11). Of these surgical site infection subtypes, the superficial incisional SSI appears to be the most challenging complication to capture correctly by classical NLP approaches (12).

We hypothesized that DL models could detect superficial surgical site infections (SSSIs) occurring within 30 days of a primary surgical procedure with a fidelity comparable to manual curation, and superior to both administrative data and previously published non-DL methods.

## Methods

### Data source

The study was approved by the Danish Patients Safety Board (Styrelsen for Patientssikkerhed, approval #31-1521-182) as well as the Danish Capital Region Data Safety Board (Videncenter for dataanmeldelser, approval #P-2020-180). In accordance with Danish law, patient consent was not required as the study was purely retrospective and without patient interaction.

This study was performed on data from a cohort of surgical patients from the Capital Region of Denmark, from January 1st, 2017, to December 31st, 2021. The Capital Region hospitals provide free of charge healthcare to approx. 1.8 million Danish citizens across 11 somatic hospitals, including care for postoperative complications. For this study, only patients with a surgical procedure performed at one of these 11 public hospitals during the study period were included. We included all surgeries from each of the 11 major surgical specialties (orthopedic surgery, general surgery, gynecology and obstetrics, urology, cardiothoracic surgery, ophthalmic surgery, plastic surgery, neurological surgery, otorhinolaryngology, oral and maxillofacial surgery, and vascular surgery).

### Administrative data

We used a combination of procedure codes identifying relevant reoperations and ICD-10 codes to identify SSSIs. The Danish coding system employs ICD-10 codes for diagnosis coding, whereas surgical procedures are registered according to the Nordic Medico-Statistical Committee (NOMESCO) Classification of Surgical Procedures (NCSP). All codes are logged by the treating physicians either during treatment or at the time of discharge. We included codes logged up to 30 days after a surgical procedure in any of the Capital Region hospitals, thus enabling us to capture SSSIs identified at both the hospital performing the initial procedure but also at other regional hospitals in case the patient was discharged from the primary hospital and treated in another hospital for the SSSI within the 30-day postoperative timeframe.

ICD-10 diagnoses codes were divided into high-confidence SSSI codes that described a postoperative wound infection, and low-confidence codes that described general skin infections in the postoperative period.

High-confidence codes included “surgical wound abscess” (DT814A), “postoperative wound infection” (DT814F), and “postoperative superficial wound infection” (DT814G). Low-confidence codes included “cutaneous abscess, furuncle, and carbuncle” (L02), “cellulitis” (L03), “local infection of the skin and subcutaneous tissue, unspecified” (L08.8), and “local infection of the skin and subcutaneous tissue, unspecified” (L08.9).

### Manual curation

Surgical cases with either a reoperation for superficial infection after surgery (NCSP code KXWB) or a high/low-confidence ICD-10 code from the above-mentioned list within 30 days of a surgical procedure were flagged for manual review. Only reoperation codes and high-confidence ICD-10 codes were used to compare performance with the NLP algorithms.

Postoperative chart notes were defined as medical free text notes logged from the time of the surgical procedure to 30 days after and included admission notes, operative notes, progress notes, radiology reports, and discharge notes. The notes were manually curated for the presence of an SSSI by a trained team of medical reviewers.

For the test dataset, all cases were re-reviewed by an external physician. The inter-rater reliability was assessed by the kappa statistic. Test cases with inter-rater disagreement were reviewed by a third-party general surgeon.

### Rule-based labeling

For the training data only, chart notes logged within the first two days of the postoperative procedure were logged as not describing an SSSI, due to the very unlikely case of an SSSI presenting in this early timeframe. This was defined as rule-based labeling and was only done for the training data.

### SSSI definition

We used the ACS NSQIP definition of SSSIs, which aligns with the CDCs definitions as stated above. Specifically, the ACS NSQIP defines SSSI's as a postoperative infection involving the skin or subcutaneous tissue of the incision occurring within 30 days after a primary surgical procedure. Additionally, one of three criteria should be met: purulent drainage, a positive culture from the superficial incision, or the diagnosis of an SSSI by a physician. For this study, a positive culture was registered if a physician chart note detailing this was present.

### Training data

**Figure 1:**
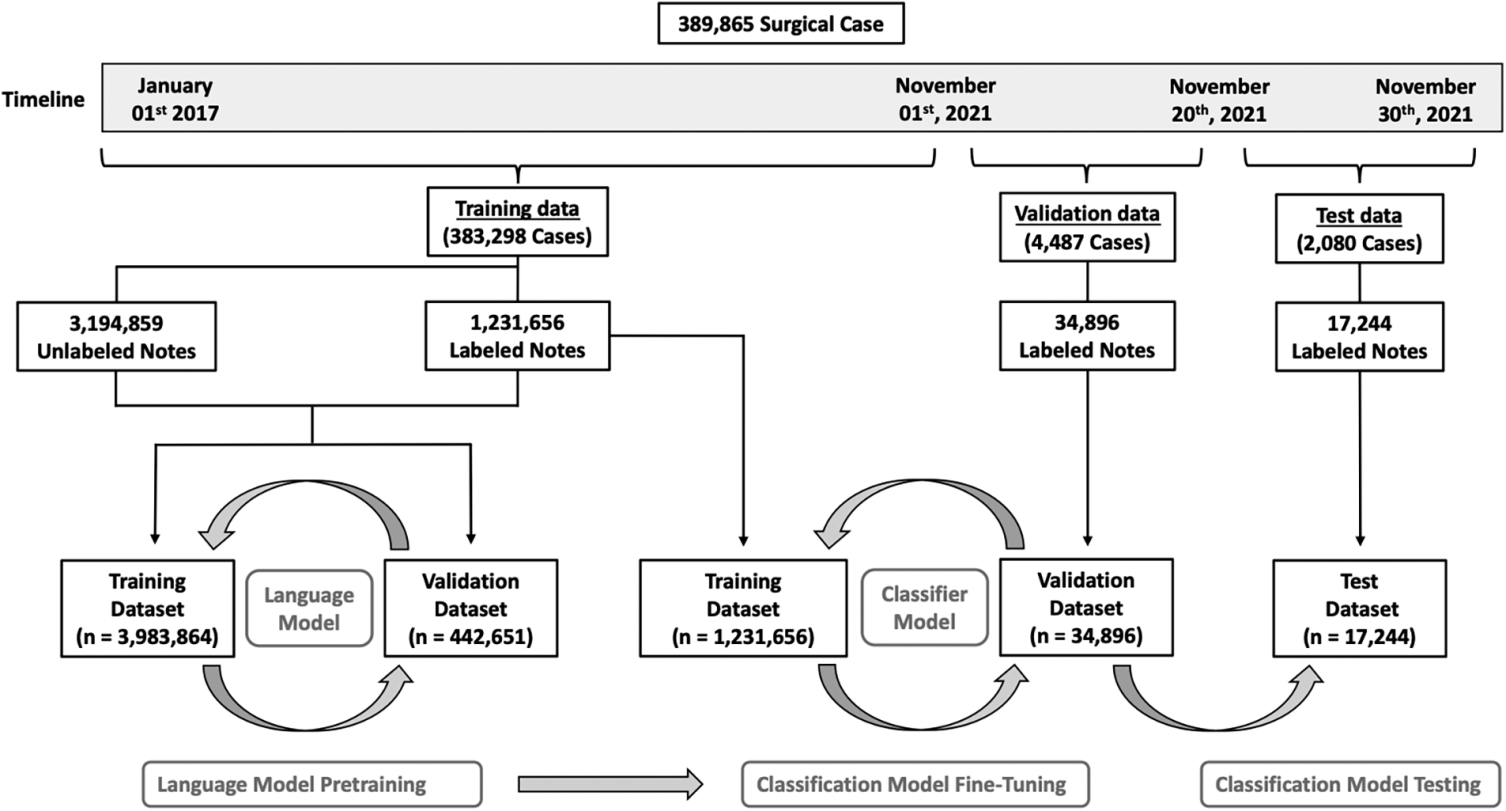
Overview of patient allocation. A total of 389,865 surgical cases were included in this study. To train the language models, we selected all notes recorded before November 2021 (*n* = 4,521,611) from a total of 383,298 surgical cases. Duplicated notes were removed ((*n* = 95,096) for a final dataset of 4,426,515 unique notes. Of these notes, 1,231,656 had a label, and 3,194,859 were unlabeled, however, labels were not used for the language models. This dataset was randomly split into a training dataset (*n* = 3,983,864) and a validation dataset (*n* = 442,651). After successfully pretraining both language models, we added a classifier to each and fine-tuned the classification models on the labeled notes from the dataset (*n* = 1,231,656). Models were validated on the 4,487 cases operated between the 1st and the 20th of November 2021. This dataset consisted of a total of 34,896 labeled notes. Finally, the classification models were tested on 2,080 cases operated between the 20th and the 30th of November 2021. This final dataset consisted of 17,244 labeled notes.

Figure 1 illustrates how the data was split between training, validation, and testing. We chose a time-based data split to simulate retrospective algorithm development with prospective algorithm testing. To maximize the amount of utilized data, we split the data on the 1st of November 2021. This allowed us to validate and test the algorithms on every patient that underwent surgery during an entire month (November 2021) at any of the 11 regional hospitals, with complete 30-day follow-up data on all patients, reaching to the end of December 2021. To train the NLP algorithms we used data recorded before the 1st of November 2021. We manually curated the subset of cases that had either a relevant reoperation code or ICD-10 code within 30 days of the surgical procedure (see definitions above).

### Validation and test data

All included chart notes from patients undergoing surgery in November 2021 (Figure 1), were manually curated for the presence of an SSSI. For this dataset, we excluded all non-primary procedures, defined as procedures performed within 30 days after an unrelated primary surgical procedure. As an example, if a patient underwent a cholecystectomy and then a hip replacement within a three-week timeframe, only the cholecystectomy was assessed. For validation and testing, we again chose a time-based split on the 20th of November 2021, so that all cases performed during the first 20 days of the month functioned as a validation dataset, and the last 10 days functioned as a test dataset.

### Data leakage

To limit the risk of data leakage from training to validation/test data, we excluded all chart notes that were recorded after October 30th, 2021, from the training dataset. As an additional quality control, we double-checked that no chart notes were present in both the training- and the validation/test datasets.

### Model architecture

We chose the Universal Language Model Fine-Tuning (ULMFiT) DL approach as proposed by Howard et al (13). The basic idea behind ULMFiT is to pretrain a universal language model (LM) on unlabeled data, and then to fine-tune it on labeled data for downstream tasks. The approach can be further optimized by ensembling a forward and a backward model into one final bidirectional classifier.

We pretrained the two language models on all postoperative chart notes recorded before November 01st, 2021 (one forward reading model and one backward reading model) (Figure 1). To limit the risk of overfitting, we excluded all duplicated notes defined as notes having identical text to another note. The data were randomly split into an LM training (90%) and an LM validation (10%) dataset. We trained a 3-layer AWD-LSTM model (Average-Stochastic Gradient Descent Weight-Dropped Long Short-Term Memory architecture) to predict the next word in the medical texts (14). The model was trained for 10 epochs, with a batch size of 128, mixed precision training, a learning rate of 2e-4, and tuned dropout hyperparameters, as presented in the original manuscript. Models were evaluated using the accuracy and perplexity performance metrics.

After successfully pretraining both language models, we added a classifier to each. Then the two models were fine-tuned on the labeled training dataset. Each classification model was trained with discriminative fine-tuning, slanted triangular learning rates, and gradual unfreezing as previously described (13). We trained both classification models for a total of 4 epochs. After each epoch, the model was validated on chart notes from surgical cases performed during the first 20 days of November 2021. Results from the validation data were used for threshold selection.

We tested the final model performance on primary surgical cases from the last 10 days of November 2021. This was done by averaging the prediction of the forward and backward model for each of the postoperative notes. If a case had at least one note, with an average model prediction above the identified threshold (see below), the case was categorized as having an SSSI.

### Threshold selection

As the classifiers output probabilities rather than classes, it is important to select an appropriate threshold for mapping probabilities to classes (i.e., mapping the probability given by the model that an SSSI occurred based on the NLP analyses to a binary yes/no classification of an SSSI). We propose two use cases: a stand-alone ML pipeline and a human-in-the-loop pipeline.

For stand-alone applications, we trained a stand-alone model (SAM). Here, false positives are costly, as none of the cases are reviewed by humans. For this use case, threshold selection can be based on optimizing the F05 score. This score gives more weight to the positive predictive value (PPV) than to sensitivity, minimizing the number of false positives at the expense of more false negatives (15).

For human-in-the-loop applications, we trained a human-in-the-loop (HITL) model. For practical use in a real-world setting, all positive cases would be manually double-checked by a human. For this use case, threshold selection was based on optimizing the F2 score. Unlike the F05 score, the F2 score gives more weight to sensitivity than to the PPV (15).

We calculated the F05 and F2 scores for thresholds from 0 to 1 in increments of 0.01, enabling the identification of the optimal threshold for each score.

### Performance

The model performance on individual notes was established using the area under the curve (AUC). On a case level, the AUC was established based on the highest individual note prediction for each case. Additional accuracy parameters on a case level included sensitivity, specificity, PPV, and negative predictive value (NPV). Both administrative data (SSSI reoperation codes and high-confidence ICD-10 codes) and NLP models were compared to manual curation.

### Data presentation

Data are presented as medians [interquartile range] or percentages where appropriate.

### Implementation

Data were processed in a secure cloud environment on Windows Azure. NLP algorithms were implemented using Python v.3.8.13, Torch v.1.13.0, and Fastai v.2.7.10. We calculated the performance metrics using the Scikit Learn python package v.1.1.3.

## Results

The overall dataset consisted of 389,865 surgical cases (296,764 patients) and 4,573,751 chart notes, of which 135,508 notes were manually curated. In the overall patient cohort, the median age was 58 [36–72], the median operation time was 54 min [29–91] and 54.4% of patients were female. The distribution of cases across the surgical specialties and procedure subtypes is presented in the online Supplementary Material (Table S1 and S2). An overview of patient allocation is presented in Figure 1.

### Language models

To train the two language models, we selected all notes recorded before November 2021 (*n* = 4,521,611) from a total of 383,298 surgical cases. We then removed duplicated chart notes (*n* = 95,096) for a final unlabeled dataset of 4,426,515 unique notes. The median word count of the notes was 92 [40–198]. This dataset was then split randomly into an LM training dataset (*n* = 3,983,864) and an LM validation dataset (*n* = 442,651). (Figure 1).

The forward language model was able to predict the next word in the postoperative notes of the LM validation dataset with an accuracy of 59.7% and a perplexity of 7.62. The backward model achieved an accuracy of 60.7% and a perplexity of 7.89.

### Classification models

For the NLP classification of SSSIs, the training dataset consisted of 383,298 primary surgical cases with 1,231,656 labeled chart notes. Summary statistics from manual curation are presented in Table 1.

**Table 1:** Summary statistics from manual curation.

	Training data	Validation data	Test data
Case level			
Number of surgical cases	383,298	4,487	2,080
Number of manually curated cases	5,274	4,487	2,080
–Positive on manual curation	3,032 (57.5%)	108 (2.4%)	48 (2.3%)
Reoperations	307	2	2
–Positive on manual curation	235 (76.5%)	2 (100.0%)	1 (50.0%)
High-confidence ICD codes	3,277	24	10
–Positive on manual curation	2,300 (70.2%)	19 (79.2%)	5 (50.0%)
Low-confidence ICD codes	1,839	10	1
–Positive on manual curation	632 (34.4%)	5 (50.0%)	0 (0.0%)
Note level			
Number of chart notes	1,231,656	34,896	17,244
Number of notes with rule-based labels	1,148,288	0	0
Number of manually curated chart notes	83,368	34,896	17,244
–Positive on manual curation	7,825 (0.9%)	249 (0.7%)	136 (0.8%)

A total of 5,274 cases met at least one of the three criteria for manual review. Of these cases, 3,032 had an SSSI upon manual review. For training, a total of 83,368 individual postoperative notes were manually curated, of which 7,825 described an SSSI. Model performance was validated on 4,487 surgical cases, of which 108 (2.4%) had an SSSI upon manual review. This dataset consisted of 34,896 notes, of which 249 (0.7%) described an SSSI. The forward and backward classification models achieved a combined validation AUC of 0.994. The threshold that optimized the F05 score for the SAM model was 0.42, and for the F2 score (HITL model), it was 0.18.

The test dataset consisted of 2,080 surgical cases, of which 48 (2.3%) had an SSSI. On this dataset, there was an inter-rater disagreement between the trained team of medical reviewers and the medical doctor on 27 cases (1.3%), resulting in a Cohen's kappa coefficient of 0.73. These 27 cases were reviewed by a general surgeon, who classified 11 as describing an SSSI. The final test dataset consisted of 17,244 notes, of which 136 (0.8%) described an SSSI.

**Figure 2:**
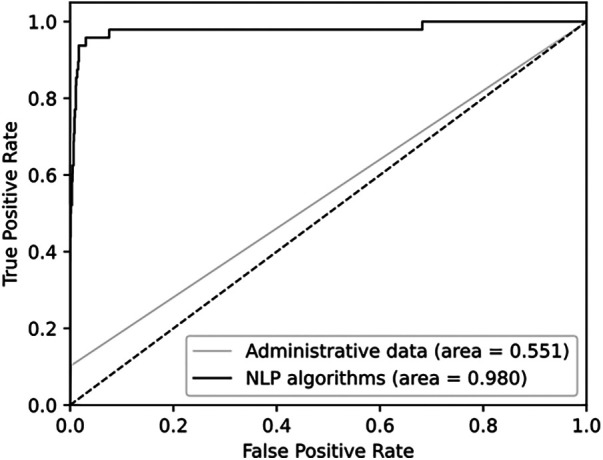
Receiver operating characteristics for administrative data and for NLP algorithms. Both are compared to manual curation and presented on a case level.

**Figure 3:**
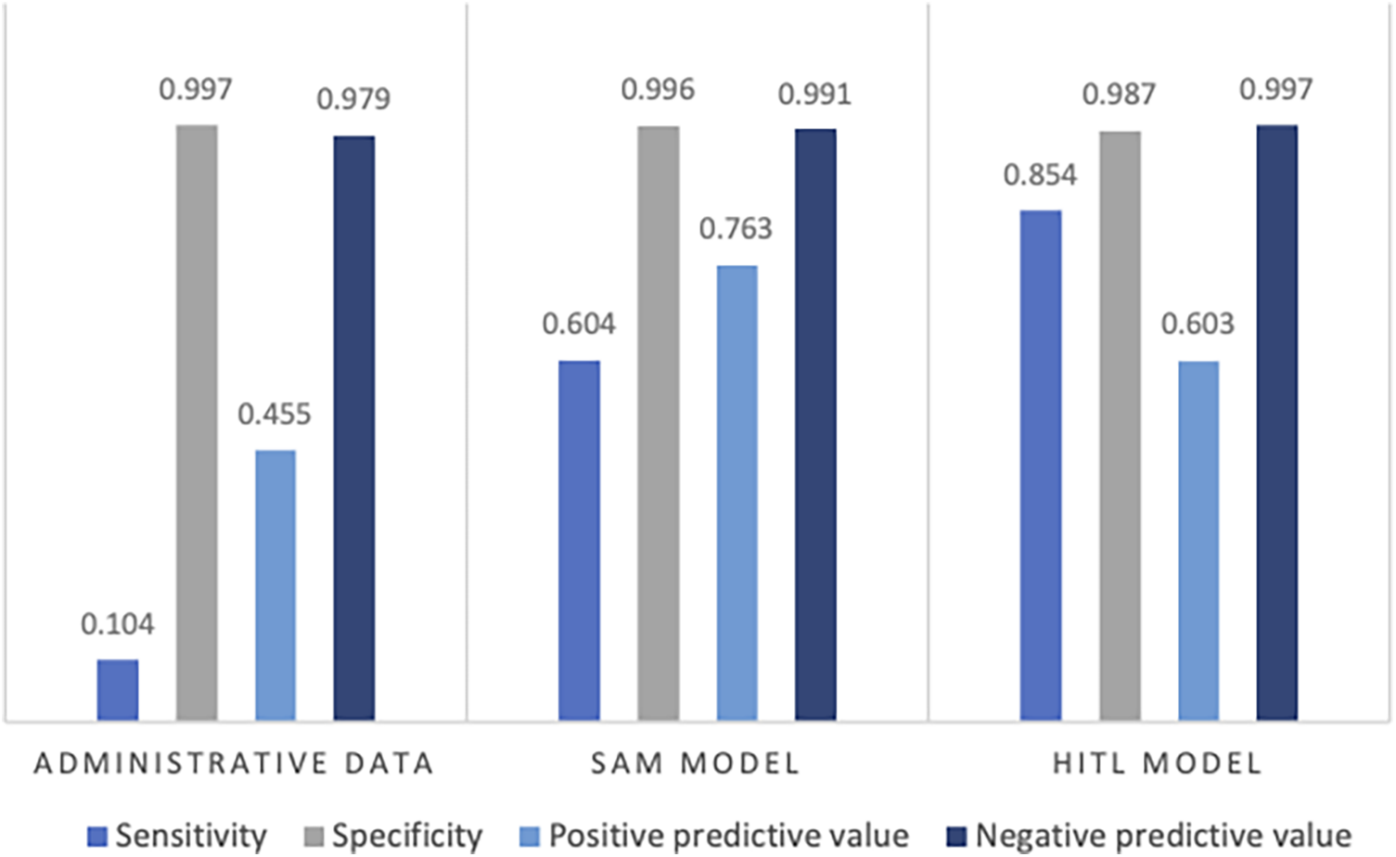
Performance metrics on the test data for administrative data, the stand-alone model (SAM), and the human-in-the-loop (HITL) models, when compared to human curation. Presented as the sensitivity, specificity, positive predictive value, and negative predictive value.

**Figure 4:**
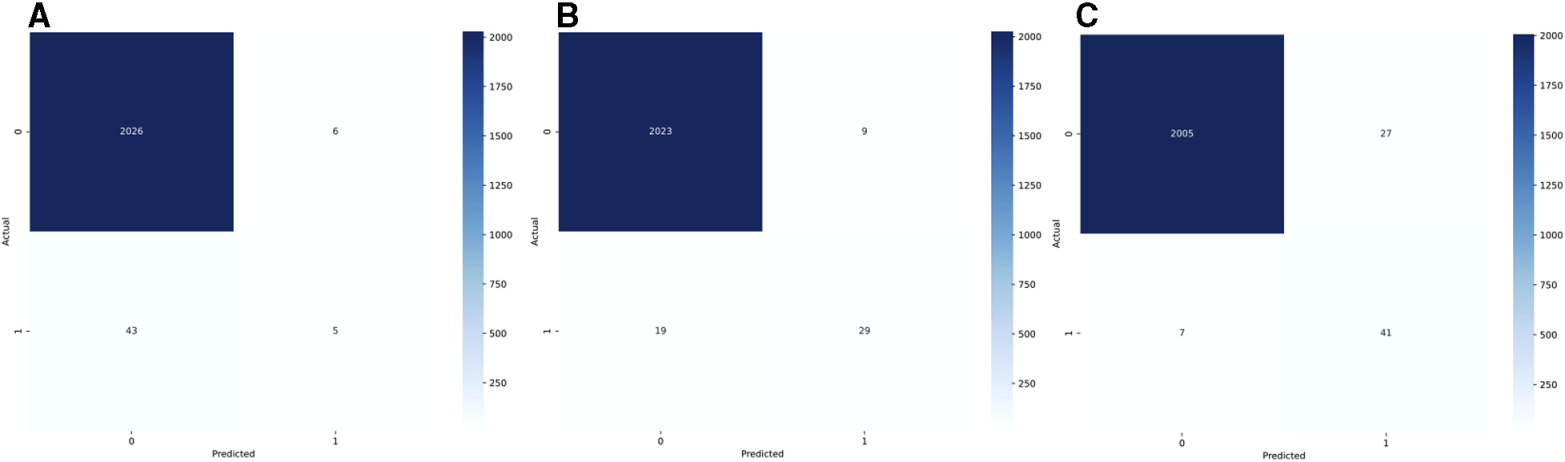
Confusion matrices for administrative data (**A**, reoperations and high-confidence ICD-10 codes), for the stand-alone model (**B**, SAM, optimized for the F05 score), and for the human-in-the-loop (**C**, HITL, optimized for the F2 score). The actual label is based on the manual curation of patient charts.

The combined test AUC was 0.989 on individual notes and 0.980 on a case level, compared to a test AUC of 0.551 for administrative data (Figure 2). On subgroup analysis, AUC scores across surgical specialties with more than one SSSI in the test set, varied from 0.978 for plastic surgery and 1.000 for neurological surgery (online Supplementary Material, Figure S1). Comparative bar graphs of SSSI cases identified by manual curation, administrative data, and the SAM model, for both the validations and the test datasets, are presented in online Supplementary Material, Figure S2. Performance metrics are graphically depicted in Figure 3. When compared to manual curation, administrative data had a sensitivity of 0.104, a specificity of 0.997, a PPV of 0.455 and a NPV of 0.979. When optimizing for the F05 score, the SAM model had a sensitivity of 0.604, a specificity of 0.996, a PPV of 0.763 an NPV of 0.991. When optimizing for the F2 score, the HITL model achieved a sensitivity of 0.854, a specificity of 0.987, a PPV of 0.603, and a NPV of 0.997. Confusion matrices are presented in Figure 4.

## Discussion

In this study, DL models were trained to detect SSSIs from unstructured free medical text. We propose two practical use cases: a stand-alone ML pipeline and a human-in-the-loop pipeline.

For the stand-alone ML pipeline, a threshold of 0.42 optimized the F05 score. This pipeline could address some of the shortcomings of administrative data with an improved sensitivity (0.604 vs. 0.104), PPV (0.763 vs. 0.455), and NPV (0.991 vs. 0.979), while retaining a similar specificity (0.996 vs. 0.997). Another advantage of this pipeline would be increased temporal precision, as the date of a positive note could easily be extracted.

For the human-in-the-loop pipeline, a threshold of 0.18 optimized the F2 score. This pipeline could further improve results, by having a human review of positive cases. By manually curating 68 out of the 2,080 cases in the test set (3.3%), it could theoretically achieve a specificity of 1, a sensitivity of 0.907, an NPV of 0.997, and a PPV of 1. For additional improvements, the threshold could be decreased further, but this would come at the expense of an increased burden of manual curation. This pipeline could address some of the shortcomings of traditional manual curation, as it would be less time-consuming, cheaper, less prone to human error, and potentially with a faster data release. Ideally, these metrics should be assessed considering reported SSSI rates in Denmark. SSSIs are, however, outside of specific research projects solely tracked through administrative codings which as seen in table 3 only retains a 10% sensitivity for capturing SSSIs. As such, there is presently no credible national overview of SSSI rates.

Many studies have investigated the performance of NLP algorithms for detecting surgical site infections, however, to our knowledge, only two studies have reported performance metrics for superficial surgical site infections, specifically (1, 12).

In the first study, Bucher et al. developed a rule-based NLP system to detect SSIs (12). Their training data included 97 SSSI cases, and they got an internal validation AUC of 0.887. For comparison, our algorithms achieved an AUC of 0.980.

In the second study, Skube et al. used a combination of structured and unstructured data, with a keyword-based NLP approach (1). Their training data included 174 SSSI cases, and they chose a threshold of 0.06. They reported a specificity of 0.879 and a sensitivity of 0.822. However, they did not report the AUC, PPV, or NPV for this model. On imbalanced data, such low thresholds (0.06) tend to increase the sensitivity and the specificity, while decreasing the PPV substantially. For comparative purposes, we saw a sensitivity of 0.958 and a specificity of 0.969 with a similar threshold of 0.06, but the PPV decreased to 0.422.

In summary, DL models from this study appear to outperform previously published non-DL results for the automatic detection of SSSIs. One possible reason for the superior performance of the presented algorithms is the amount of available training data. We trained models on 3,032 SSSI cases, compared to 97 and 174 cases from the two previously mentioned studies.

Another possible explanation could be the use of DL. To test this hypothesis, we used Microsoft Azures Machine Learning Studios AutoML function to train and validate different non-DL algorithms. We trained a total of 33 models on the same training- and validation datasets as previously described. The best performing non-DL algorithm was based on term frequency-inverse document frequency (TF-IDF), followed by a maximum absolute scaling and finally a gradient boosting classifier. This model was slightly inferior to the ULMFiT approach, with a test AUC of 0.981 (vs. 0.989 for ULMFiT) on individual notes and 0.963 (vs. 0.980 for ULMFiT) on a case level. However, the true potential of DL may be more pronounced in scenarios involving even larger datasets. It is conceivable that as the size and complexity of the dataset increase, the advantages of DL models, especially in terms of their ability to capture nuanced patterns and relationships in data, become more evident. This hypothesis warrants further investigation and could form the basis for future studies.

One advantage of our approach was the flagging of training cases for manual curation. The presented results were achieved by manually curating under 2% (83,368) of the available 4,426,515 postoperative notes in the training dataset.

Another advantage was the use of individual notes instead of aggregated notes. This resulted in a higher burden of manual review for training, but increased the positive training documents from 3,032 aggregated documents (one for each SSSI case) to 7,852 positive individual notes. This approach also allowed us to include many auto-labeled negative notes in the training dataset, increasing the number of negative notes from 75,543 to 1,223,831. It also provided insights into the classification of each note, allowing for a rapid review of positive cases/notes, and potentially also insights into the phrases that drive predictions.

### Limitations

One limitation of this study is the challenge of establishing a ground truth. In some cases, ICD-10 codes could represent the ground truth, and qualify as one of the SSSI criteria (Diagnosis of SSSI by a physician), even though the postoperative notes did not describe an SSSI. However, during manual curation, we saw multiple examples of positive ICD-10 codes, where the postoperative notes explicitly stated, that the patient did not have an SSSI. For this reason, we chose to use manual curation as the ground truth, instead of a combination of manual curation and positive codes.

In Denmark, most postoperative complications are treated in hospitals, and we, therefore, expect to have relevant clinical follow-up data on most patients. However, mild SSSIs could potentially be treated by a general practitioner, and these cases would not appear in the hospital EHR. This further underlines the important point of clinical relevance. While SSSI's are clinically important, the severity may vary widely between cases with some cases requiring hospital admission and others handled in the outpatient setting. Future studies could thus ideally focus on using DL NLP approaches to assess the severity and not just the presence of SSSI's.

Finally, it should be noted that variations in SSSI rates between the training, validation and test sets could be present. This would, however, only effect the amount of SSSI cases available for model training, as the final performance is assessed only on the test set.

## Conclusion

In this study, DL models were pretrained on 4,426,515 unlabeled postoperative notes and fine-tuned to detect SSSIs on 1,231,656 labeled notes. Overall, the models appeared to outperform previously published results. As a stand-alone ML pipeline, models outperformed administrative data, while theoretically retaining key advantages such as easy accessibility, inexpensive capture, and improved temporal precision. Deployed as a human-in-the-loop pipeline, performance metrics approach that of manual curation while being cheaper, less time-consuming, and with a potential for rapid data release.

## Data Availability

The underlying dataset is available upon institutional review board approval from the Capital Region of Denmark (videncenter for regional udvikling) as well as approval from the Data Safety Unit of the Capital Region of Denmark. Due to patient confidentially issues, the authors are not allowed to share the dataset without written approval from the above-mentioned governing bodies. The underlying code for this study can be shared on reasonable request. Due to intellectual property issues, parts of the codes and generated networks are not available for sharing. Please contact the corresponding author(s) for further enquiries.

## References

[1] Skube SJ Hu Z Simon GJ Wick EC Arsoniadis EG Ko CY Accelerating surgical site infection abstraction with a semi-automated machine-learning approach. Ann Surg. (2022) 276(1):180–5. 10.1097/SLA.0000000000004354 33074897

[2] Murff HJ FitzHenry F Matheny ME Gentry N Kotter KL Crimin K Automated identification of postoperative complications within an electronic medical record using natural language processing. JAMA. (2011) 306(8):848–55. 10.1001/jama.2011.1204 21862746

[3] Chapman AB Mowery DL Swords DS Chapman WW Bucher BT. Detecting evidence of intra-abdominal surgical site infections from radiology reports using natural language processing. AMIA Annu Symp Proc. (2017) 2017:515–24. 29854116 PMC5977582

[4] Hollenbeak CS Boltz MM Wang L Schubart J Ortenzi G Zhu J Cost-effectiveness of the national surgical quality improvement program. Ann Surg. (2011) 254(4):619–24. 10.1097/SLA.0b013e318230010a 22039608

[5] Morris MX Song EY Rajesh A Kass N Asaad M Phillips BT. New frontiers of natural language processing in surgery. Am Surg. (2023) 89(1):43–8. 10.1177/00031348221117039 35969539

[6] Esteva A Robicquet A Ramsundar B Kuleshov V DePristo M Chou K A guide to deep learning in healthcare. Nat Med. (2019) 25(1):24–9. 10.1038/s41591-018-0316-z 30617335

[7] Mellia JA Basta MN Toyoda Y Othman S Elfanagely O Morris MP Natural language processing in surgery: a systematic review and meta-analysis. Ann Surg. (2021) 273(5):900–8. 10.1097/SLA.0000000000004419 33074901

[8] Gori D Banerjee I Chung BI Ferrari M Rucci P Blayney DW Extracting patient-centered outcomes from clinical notes in electronic health records: assessment of urinary incontinence after radical prostatectomy. EGEMS (Wash DC). (2019) 7(1):43. 10.5334/egems.297 31497615 PMC6706996

[9] Anteby R Soffer S Nachmany I Klang E. Comment on “natural language processing in surgery: a systematic review and meta-analysis”. Ann Surg. (2021) 274(6):e941–e2. 10.1097/SLA.0000000000004939 34016811

[10] Etzioni DA Lessow CL Lucas HD Merchea A Madura JA Mahabir R Infectious surgical complications are not dichotomous: characterizing discordance between administrative data and registry data. Ann Surg. (2018) 267(1):81–7. 10.1097/SLA.0000000000002041 27759619

[11] Horan TC Andrus M Dudeck MA. CDC/NHSN surveillance definition of health care-associated infection and criteria for specific types of infections in the acute care setting. Am J Infect Control. (2008) 36(5):309–32. 10.1016/j.ajic.2008.03.002 18538699

[12] Bucher BT Shi J Ferraro JP Skarda DE Samore MH Hurdle JF Portable automated surveillance of surgical site infections using natural language processing: development and validation. Ann Surg. (2020) 272(4):629–36. 10.1097/SLA.0000000000004133 32773639 PMC9040555

[13] Howard J Ruder S. Universal language model fine-tuning for text classification. arXiv preprint arXiv:180106146. 2018.

[14] Merity S Keskar NS Socher R. Regularizing and optimizing LSTM language models. arXiv preprint arXiv:170802182. 2017.

[15] Sasaki Y. The truth of the F-measure. Teach Tutor Mater. (2007) 1(5):1–5.

