# Supplementary material for: Assessing the utility of deep neural networks in detecting superficial surgical site infections from free text electronic health record data
Source: Front Digit Health. 2024 Jan 8;5:1249835. doi: 10.3389/fdgth.2023.1249835 (PMC10801170; doi:10.3389/fdgth.2023.1249835)
Supplement: Supplementary file 1 [file Table1.docx]

**SUPPLEMENTARY TABLES**

**Table S1**

The distribution of cases across the surgical specialties included in this study.
SSSI: Superficial Surgical Site Infection

| **Surgical specialty** | **Number of surgical cases** | | | **SSSI Incidence** | |
| --- | --- | --- | --- | --- | --- |
|  | **Training data** | **Validation data** | **Test data** | **Validation data** | **Test data** |
| Orthopedic surgery | 101,748 | 1,070 | 515 | 17 (1.6%) | 6 (1.2%) |
| General surgery | 72,519 | 822 | 418 | 26 (3.2%) | 11 (2.6%) |
| Gynecology and obstetrics | 42,114 | 510 | 241 | 14 (2.7%) | 6 (2.5%) |
| Urology | 29,217 | 367 | 158 | 8 (2.2%) | 1 (0.6%) |
| Cardiothoracic surgery | 26,812 | 236 | 101 | 10 (4.2%) | 9 (8.9%) |
| Ophthalmic surgery | 24,739 | 522 | 219 | 1 (0.2%) | 1 (0.5%) |
| Plastic surgery | 21,245 | 277 | 140 | 13 (4.7%) | 9 (6.4%) |
| Neurological surgery | 19,792 | 184 | 77 | 3 (1.6%) | 2 (2.6%) |
| Otorhinolaryngology | 18,172 | 209 | 88 | 0 (0.0%) | 0 (0.0%) |
| Oral and maxillofacial surgery | 13,635 | 118 | 53 | 3 (2.5%) | 1 (1.9%) |
| Vascular surgery | 13,305 | 172 | 70 | 13 (7.6%) | 2 (2.9%) |
| **Total** | **383,298** | **4487** | **2080** | **108 (2.4%)** | **48 (2.3%)** |

**Table S2**

Distribution of superficial surgical site infections (SSSIs) between 10 surgical procedure subtypes where SSSIs were most prevalent.

| **Surgical procedures** | **Number of procedures** | **Number of SSSIs** |
| --- | --- | --- |
| Exploratory laparotomy | 34 | 7 (20.6%) |
| Excision of pathological tissue in the skin and subcutaneous tissue of the head | 60 | 5 (8.3%) |
| Thoracoscopic wedge resection of the lung | 24 | 4 (16.7%) |
| Thoracoscopic lobectomy of the lung | 22 | 4 (18.2%) |
| Placement of pleural drain | 31 | 3 (9.7%) |
| Primary insertion of the distal component of an uncemented partial hip prosthesis | 59 | 3 (5.1%) |
| Cesarean section in the isthmus of the uterus during childbirth due to birth complication | 100 | 3 (3.0%) |
| Incision of the skin on the trunk | 28 | 3 (10.7%) |
| Excision of pathological tissue in the skin and subcutaneous tissue of the lower extremity | 34 | 3 (8.8%) |
| Planned cesarean section in the isthmus of the uterus before childbirth | 115 | 3 (2.6%) |

**SUPPLEMENTARY FIGURES**

**Figure S1.** Receiver Operating Characteristics for NLP algorithms on each surgical specialty with more than one Superficial Surgical Site Infection (SSSI) case in the test dataset. Performance is compared to manual curation and presented on a case level.

**Figure S2.** A comparative bar graph of Superficial Surgical Site Infection (SSSI) cases identified by manual curation, administrative data, the Stand-alone model (SAM), and the Human-in-the-loop (HITL) Model for both the validation and the test data. Each bar illustrates true positives (dark grey) and false positives (light grey). The y-axis represents the number of cases.
