# Supplementary figures and images for: Assessing the utility of deep neural networks in detecting superficial surgical site infections from free text electronic health record data
Source: Front Digit Health. 2024 Jan 8;5:1249835. doi: 10.3389/fdgth.2023.1249835 (PMC10801170; doi:10.3389/fdgth.2023.1249835)

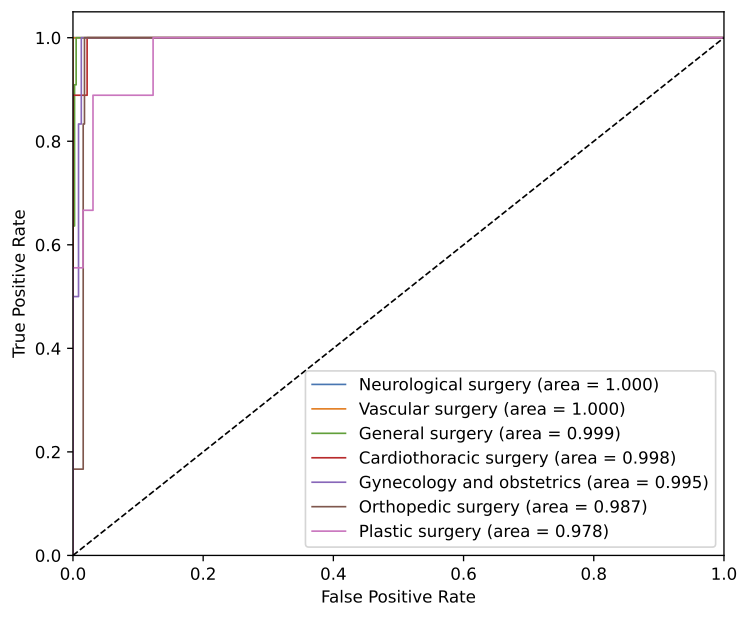

Supplement: Supplementary file 2 [file Image1.tiff]

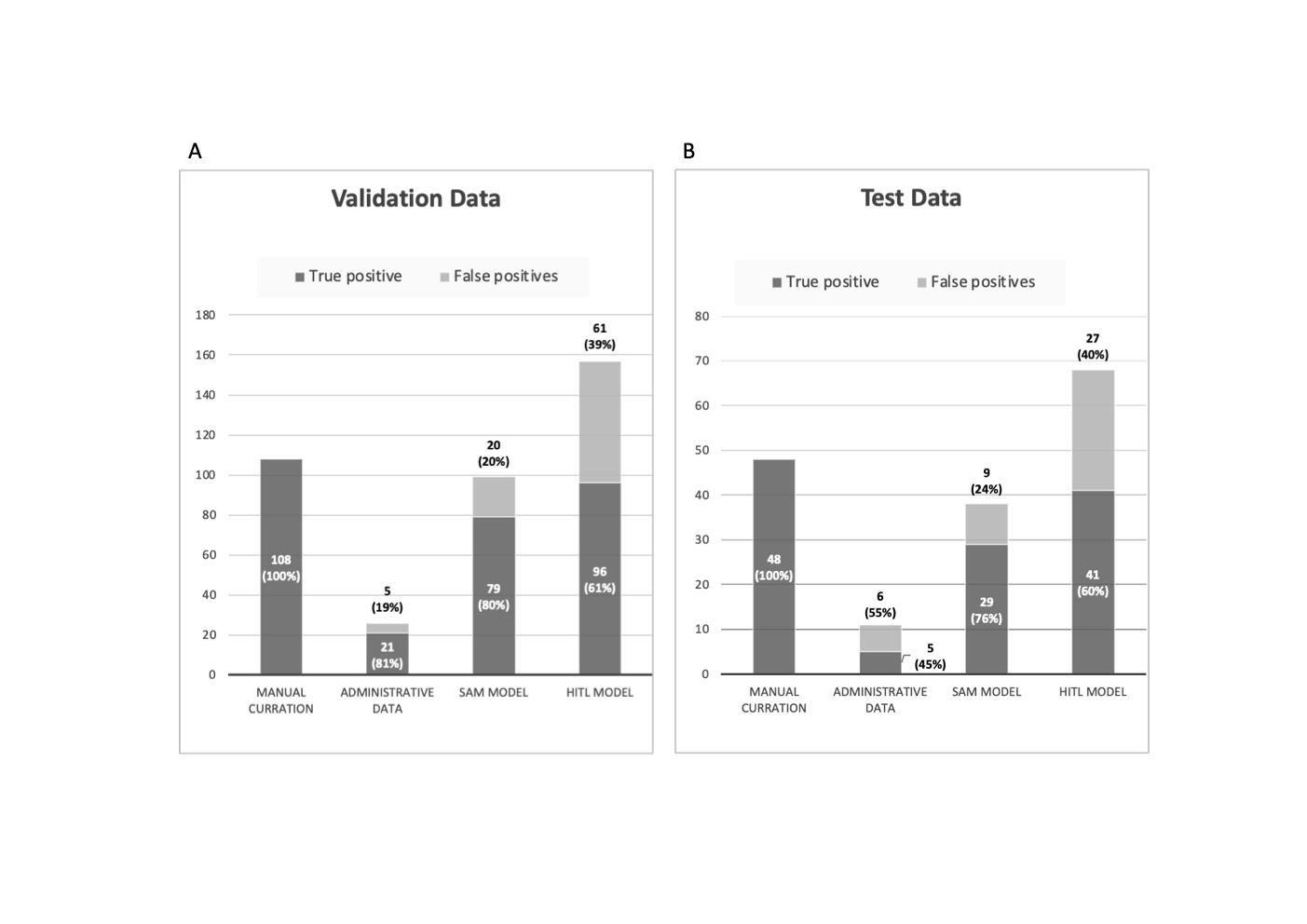

Supplement: Supplementary file 3 [file Image2.tiff]
